# Alpha/beta values in pediatric medulloblastoma: implications for tailored approaches in radiation oncology

**DOI:** 10.1186/s13014-024-02566-8

**Published:** 2025-01-29

**Authors:** Danny Jazmati, Dennis Sohn, Juliane Hörner-Rieber, Nan Qin, Edwin Bölke, Jan Haussmann, Rudolf Schwarz, Niklas David Niggemeier, Arndt Borkhardt, Florian Babor, Triantafyllia Brozou, Melissa Felek, Balint Tamaskovics, Patrick Melchior, Thomas Beez, Beate Timmermann, Marc Remke, Stefanie Corradini, Rémi Till Schulz, Ronja-Linda Preugschas, Wilfried Budach, Christiane Matuschek

**Affiliations:** 1 Department of Radiotherapy and Radiooncology, Medical Faculty, Heinrich Heine University, Moorenstr. 5, 40225 Dusseldorf, Germany; 2 Laboratory of Molecular Radiooncology, Clinic and Policlinic for Radiation Therapy and Radiooncology, Medical Faculty, University Hospital Düsseldorf, Heinrich-Heine-University Düsseldorf, Düsseldorf, Germany; 3 ³Department of Pediatric Oncology, Hematology and Clinical Immunology, Medical Faculty, Center of Child and Adolescent Health, Heinrich-Heine-University, Moorenstrasse 5, 40225 Düsseldorf, Germany; 4 Department of RT and Radiooncology, Outpatient Center, University Medical Center Hamburg-Eppendorf, Martinistraße 52, 20246 Hamburg, Germany; 5 Department of Radiation Oncology, Saarland University Hospital, Homburg, Germany; 6 Department of Neurosurgery, Medical Faculty, Heinrich-Heine-University, Moorenstrasse 5, 40225 Düsseldorf, Germany; 7 Department of Particle Therapy, West German Proton Therapy Centre, University Hospital Essen, 45147 Essen, Germany; 8 Department of Pediatric Oncology and Hematology, Saarland University Medical Center, Homburg, Germany; 9 Department of Radiation Oncology, LMU University, Munich, Germany; 10 Present Address: Bielefeld University, Medical School and University Medical Center OWL, Klinikum Mitte, Department of Radiation Oncology, Bielefeld, Germany

**Keywords:** Radiation therapy, Cancer, Paediatric tumor, Brain tumor

## Abstract

**Background:**

Medulloblastoma is the most common malignant pediatric brain tumor, typically treated with normofractionated craniospinal irradiation (CSI) with an additional boost over about 6 weeks in children older than 3 years. This study investigates the sensitivity of pediatric medulloblastoma cell lines to different radiation fractionation schedules. While extensively studied in adult tumors, these ratios remain unknown in pediatric cases due to the rarity of the disease.

**Materials and methods:**

Five distinct medulloblastoma cell lines (ONS76, UW228-3, DAOY, D283, D425) were exposed to varying radiation doses and fractionation schemes. In addition, ONS76 and UW228-3 stably overexpressing MYC were analyzed. Alpha/beta values, representing fractionation sensitivity, were quantified using the linear-quadratic model of radiation survival.

**Results:**

The study unveiled elevated alpha/beta ratios across diverse medulloblastoma cell lines, with a weighted mean alpha/beta value of 11.01 Gy (CI: 5.23–16.79 Gy). Neither TP53 status nor the levels of MYC expression influenced fractionated radiosensitivity. Furthermore, differences in alpha/beta values cannot be correlated with molecular subgroups (*p* = 0.07) or radiosensitivity (SF2).

**Conclusion:**

These in vitro findings strongly recommend normofractionated or hyperfractionated radiotherapy for paediatric medulloblastoma cases due to consistently high alpha/beta values across subgroups. Conversely, hypofractionated radiotherapy is not advisable within a curative approach. This study presents significant potential by enabling the estimation of radiobiological fractionations and dose effects in young, vulnerable patients, highlighting its importance for advancing patient-specific therapeutic strategies.

## Introduction

Medulloblastoma is the most common malignant pediatric brain tumor, impacting approximately 0.5 to 1 in every 100,000 children annually [1, 2]. It is predominantly diagnosed in young patients, with a median age at onset of 5–6 years. This malignancy is characterized by its high propensity for metastasis along the neuroaxis. Additionally, approximately 30–40% of patients present with craniospinal fluid dissemination at initial diagnosis [3, 4]. The World Health Organization classifies medulloblastoma into four distinct molecular subgroups: The new WHO classification for medulloblastoma includes Wingless (WNT)-activated, Sonic Hedgehog (SHH)-activated with TP53 wild type, SHH-activated with TP53 mutation, and non-WNT/non-SHH categories [3]. These subgroups exhibit unique genetic alterations, demographic patterns, clinical behaviors, and prognostic outcomes, necessitating tailored therapeutic strategies for each subgroup [5, 6].

Treatment typically involves surgical resection, radiotherapy, and chemotherapy. Patients are stratified into standard-risk and high-risk categories based on a combination of clinical, histopathological, and cytogenetic factors. High-risk patients typically undergo an intense radiotherapy protocol, consisting of 36 Gy of craniospinal irradiation (CSI) with an additional 18 Gy boost to the posterior fossa. In contrast, standard-risk patients are treated with a less intensive CSI protocol of 23.4 Gy, delivered in fractions of 1.8 Gy [7].

Despite the effectiveness of these treatment modalities, they often lead to severe long-term side effects, including cognitive, auditory, and endocrine dysfunctions [8]. These adverse effects significantly diminish the quality of life for survivors, underscoring the critical need for therapeutic strategies that balance efficacy with minimized long-term toxicities [9]. A promising approach to achieve this balance involves the reduction of the total radiation dose.

Another strategy to modify the therapeutic window involves adjusting the dose per fraction [10, 11]. However, determining the optimal fractionation scheme in medulloblastoma remains elusive due to our limited understanding of medulloblastoma cell responses to varying fractionation schemes. The linear-quadratic model, used to estimate clinical effects of different fractionation regimens, relies on the tissue’s alpha/beta ratio [12]. Tumors with high alpha/beta ratios are typically treated with standard or hyperfractionated radiotherapy, while those with lower ratios may benefit from higher dose-per-fraction or hypofractionated radiotherapy. In this regard, an alpha/beta level is considered high if the value is above 8 Gy and low if it is below 5 Gy. A higher dose per fraction would lead to a shorter total radiation time, which could be particularly beneficial in radiation treatments for children, ultimately enhancing their quality of life during and after treatment. A comprehensive understanding of the alpha/beta ratio is crucial for optimizing the therapeutic ratio by amplifying the antitumor effect without increasing late effects. The clinical adoption of various fractionation approaches in medulloblastoma underscores a significant gap in our understanding of pediatric patients’ fractionation response. In this study, we aim to present novel experimental data on the alpha/beta ratio of medulloblastoma cells, contributing to defining dose concepts for future clinical trials in this sensitive patient population.

## Materials and methods

### Cell line cultivation and conditions

The medulloblastoma cell lines ONS76, UW228-3, DAOY, D283 and D425, representing distinct TP53 and MYC alteration profiles (Table 1), were used in this study. In addition, previously generated ONS76 and UW228-3 cells stably expressing either mCherry as a control or MYC to mimic activation of this oncogene, were employed (Table 2) [13]. ONS76, UW228-3 and DAOY cells were a kind gift from P. Landgraf (University of Cologne, Germany), whereas the D283 and the D425 cell lines were from G. Reifenberger (Heinrich-Heine University Düsseldorf, Germany). The MCF-7 mammary carcinoma cell line was obtained from the late R.U. Jänicke [14]. All medulloblastoma cell lines as well as the MCF-7 cells were authenticated by STR from the Genomics & Transcriptomic Laboratory of the Biological and Medical Research Center (BMFZ, Heinrich-Heine-university Düsseldorf, Germany), the German Collection of Microorganisms and Cell cultures (Leibniz Institute DSMZ, Braunschweig, Germany) or by the Multiplex human Cell line Authentication Test (MCA; Multiplexion GmbH, Heidelberg, Germany), respectively. ONS76, UW228-3 and DAOY cells were cultured in Dulbecco’s Modified Eagle’s Medium containing GlutaMAX, 4.5 g/l D-glucose and sodium pyruvate (DMEM, Gibco, Waltham, MA, USA), whereas the D283 and D425 cell lines were cultivated in Minimal Essential Medium containing GlutaMAX (MEM, Gibco) and modified Improved Minimal Essential Medium containing 2 mM L-glutamine (IMEM, Gibco), respectively. MCF-7 cells were grown in RPMI-1640 GlutaMAX (Gibco) containing 100 U/ml penicillin and 0.1 mg/ml streptomycin (Sigma-Aldrich, Merck Millipore, Darmstadt, Germany). All media were supplemented with 10% fetal bovine serum (FBS Supreme, PAN-Biotech GmbH, Aldenbach, Germany). The cells were incubated at 37 °C in a 5% CO_2_ atmosphere. Irradiations were performed with a Gulmay RS225 X-ray tube (Xstrahl GmbH, Ratingen, Germany) using 150 kV and 15 mA.

**Table 1:** Alpha/beta values of medulloblastoma cell lines

Cell line	Subgroup	TP53	MYC expr.	α/β +/- CI [Gy]
ONS76	SHH	wild-type	low	17.09 +/- 6.69
UW228-3	SHH	mutated	low	9.36 +/- 3.87
DAOY	SHH	mutated	low	75.09 +/- 61.66
D283	non-WNT/ non-SHH	mutated	high	15.09 +/- 16.77
D425	non-WNT/ non-SHH	mutated	high	8.42 +/- 5.85

**Table 2:** Alpha/beta values of medulloblastoma cell lines genetically modified to overexpress MYC

Cell line	Stably expr.	α/β +/- CI
ONS76	mCherry	13.69 +/- 5.21
ONS76	MYC	10.93 +/- 5.54
UW228-3	mCherry	15.82 +/- 9.40
UW228-3	MYC	19.64 +/- 10.48

### Well Control Dose Assay

The well control dose assay was adapted from experiments described previously [15]. Cell lines were seeded in 24-well-plates. In one half of the plate a high cell number was seeded (2,000 cells for ONS76, UW228-3 and D425; 4,000 cells for DAOY and D283 cells), whereas 1/10 of this number was seeded in the other half (200 and 400 cells, respectively). For the MCF-7 control cell line only the results obtained from the higher cell number (2,000) were used for analysis, as lower cell numbers were not able to display regrowth. To incorporate the impact of potential cellular cooperation effects, lethal one high dose-irradiated (20 Gy) feeder cells from the same cell line were added to the lower concentration wells to generate similar cell densities. After seeding, as soon as the cells became adherent, the fractionated irradiation was initiated. Over the span of four days, the samples were either irradiated twice a day (separated by 6–8 h), once a day or once at the beginning and once at the end of the treatment schedule, resembling 8, 4 or 2 fractions, respectively. Depending on the individual radio-sensitivity of the used cell lines, each fractionation scheme featured 5–6 different doses ranging from 0.4 to 12 Gy single dose to 3.2–32 Gy total dose. One full 24-well-plate was used for each single irradiation and fractionation condition.

**Fig. 1:**
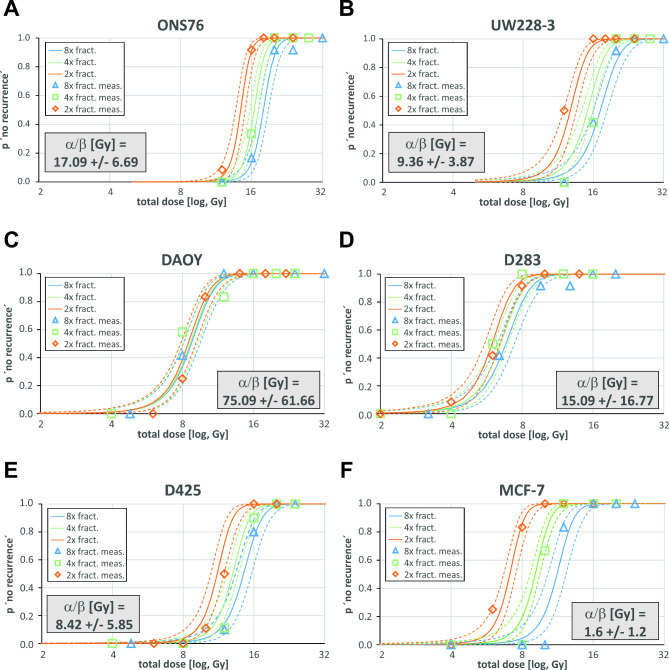
Modelled survival curves plotted against determined recurrence data of medulloblastoma cell lines Probability of recurrence after fractionated irradiation as determined by non-linear regression of survival data of the medulloblastoma cell lines ONS76 (**A**), UW228-3 (**B**), DAOY (**C**), D283 (**D**), D425 (**E**) as well as the breast cancer cell line MCF-7 (**F**) as a control with a low alpha/beta value. The solid lines depict the derived estimated survival curves for the denoted amount of cells after splitting of the full dose D (x-axis) in 2 (orange), 4 (green) or 8 (blue) fractionations. The coloured dashed lines represent the corresponding high and low 95% confidence intervals. The actual determined data for the chosen conditions (lower cell number and 2, 4, and 8 fractions) is shown as open diamond (2x fract.), square (4x fract.) and triangle (8x fract.) data points using the same colour code

Over a 60-day period, cell proliferation in each well was monitored every two to three days and scored binary for either ongoing proliferation leading to regrowth (i.e., the formation of a confluent cell monolayer) or absence of regrowth at the end of the time period. As an internal control for the Well Control Dose Assay, we employed the MCF-7 breast cancer cell line that was already reported to exhibit a low alpha/beta value far below < 8 Gy [16] which is also suggested to be generally the case for breast cancer [12]. Within our experimental setup, MCF-7 cells possess an alpha/beta ratio of 1.6 Gy +/- 1.2 (95% CI; Fig. 1F), which is in concordance with the literature.

### Statistical analysis and radiobiological modelling

The determined experimental survival probabilities were employed together with the linear-quadratic model of radiation survival,

$$\:p={e}^{-k*{e}^{-n*\left(\alpha\:*d+\beta\:*{d}^{2}\right)+\gamma\:*t}}$$

(with p = probability of well control; k = number of clonogenic cells per well; n = number of fractions; α, β = radiation sensitivity parameters; d = single dose; γ = repopulation factor; t = overall treatment time), which was transformed to

$$\:p={e}^{-{e}^{(\text{ln}\left(k\right)-\beta\:*\left(\frac{\alpha\:}{\beta\:}*D+D*d\right))}}$$

(with D = d * n, i.e. total dose)

so that the α/β value could be determined directly by modelling. ‘γ ∗ t’ was omitted because the overall treatment time for all samples was the same. Using this formula, the determined experimental recurrence probabilities and the SPSS Statistics software (Version 29.0.0.0, IBM, Armonk, NY, USA), we performed a non-linear regression with the three parameters ‘ln(k)’, ‘β‘ and ‘α/β’. A maximum likelihood estimate was employed based on a classical logarithmic loss function for binary data (-Sum (Outcome*ln(PRED_) + (1-Outcome)*ln(1-PRED_)); with Outcome = 0 for regrowth and = 1 for no recurrence at the end of the observation period) and minimization of the negative log-likelihood. Additionally, Bootstrapping was performed to estimate confidence intervals (CI). Calculations were done with SPSS statistics software package. The weighted means of the estimated alpha/beta values were calculated for wildtype and genetically modified cells lines as well as for all cell lines by using the excel-plugin MetaXL (V5.3, EpiGear.com, EpiGear Ltd) employing the inverse variance heterogeneity method. The α-parameter was calculated later on using the same method and dataset together with slightly changed formula to directly estimate ‘α’, ‘β’ and ‘ln(k)’. The obtained mean values for β and ln(k) were identical with both estimation methods, however, marginal differences in 95% CI were generated. The CI displayed in Table 3 are derived from the modelling together with ‘α/β’ estimation. The survival fraction 2 Gy (SF2) was determined using the classical LQ model and the alpha and beta values derived from our dataset.

**Table 3:** Radiation sensitivity parameter values of medulloblastoma cell lines derived from our dataset using the LQ model

Cell line	α +/- CI	β +/- CI	SF2 (calc.) +/- CI
ONS76	0.321 +/- 0.066	0.019 +/- 0.005	0.49 +/- 0.03
UW228-3	0.262 +/- 0.055	0.028 +/- 0.007	0.53 +/- 0.03
ONS76/mCherry	0.370 +/- 0.098	0.027 +/- 0.010	0.43 +/- 0.04
ONS76/c-Myc	0.326 +/- 0.095	0.030 +/- 0.009	0.46 +/- 0.05
UW228-3/mCherry	0.356 +/- 0.088	0.022 +/- 0.006	0.45 +/- 0.04
UW228-3/c-Myc	0.375 +/- 0.096	0.019 +/- 0.007	0.44 +/- 0.04
DAOY	0.501 +/- 0.103	0.007 +/- 0.006	0.36 +/- 0.04
D283	0.495 +/- 0.102	0.033 +/- 0.020	0.33 +/- 0.04
D425	0.382 +/- 0.127	0.045 +/- 0.017	0.39 +/- 0.05

## Results

**Fig. 2:**
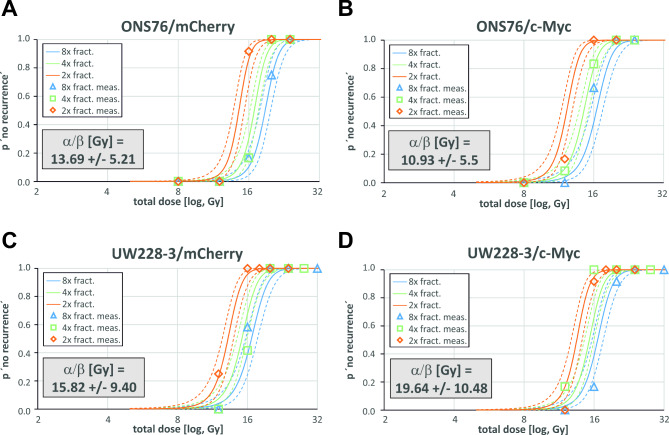
Modelled survival curves plotted against determined recurrence data of medulloblastoma cell lines genetically modified to overexpress MYC Probability of recurrence after fractionated irradiation as determined by non-linear regression of survival data of the medulloblastoma cell lines ONS76/mCherry (**A**), ONS76/MYC (**B**), UW228-3/mCherry (**C**) and UW228-3/MYC (**D**). The solid lines depict the derived estimated survival curves for the denoted amount of cells after splitting of the full dose D (x-axis) in 2 (orange), 4 (green) or 8 (blue) fractionations. The coloured dashed lines represent the corresponding high and low 95% confidence intervals. The actual determined data for the chosen conditions (lower cell number and 2, 4, and 8 fractions) is shown as open diamond (2x fract.), square (4x fract.) and triangle (8x fract.) data points using the same colour code

The tables and charts reflect the reactions of all medulloblastoma cell lines to radiation, demonstrating the variability of radiobiological responses observed. Visualization of the determined survival curves together with the actually obtained recurrence data points demonstrates its high fidelity (Figs. 1A-E and 2A-D). This study revealed high alpha/beta ratios that are all above 8 Gy across a wide range of medulloblastoma cell lines, weighted mean alpha/beta value of 11.01 Gy (CI: 5.23–16.79 Gy) (Table 1). We were able to achieve relatively small confidence intervals. The one exception is the D283 cell line that characteristically grows in a semi-adherent manner. This makes it difficult to score the wells properly, which therefore resulted in high uncertainty and bigger CI of its determined alpha/beta ratio (Fig. 1D).

**Fig. 3:**
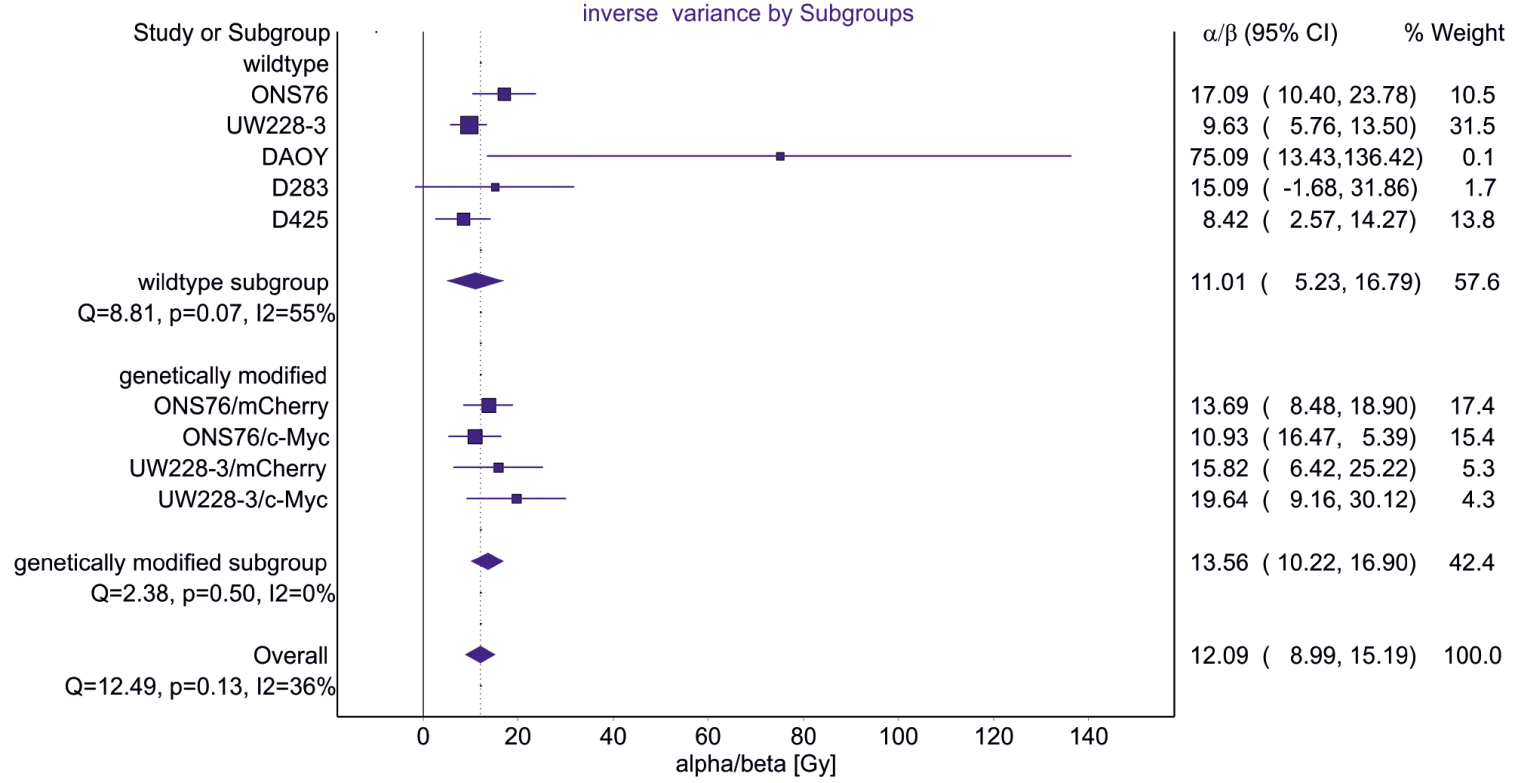
Graphical representation of the meta-analyses of the determined alpha/beta-values in medulloblastoma by MetaXL Shown is the meta-analysis of the determined alpha/beta-values in wild-type (ONS76, UW228-3, DAOY, D283, D425) and the genetically modified (ONS76/UW228-3 with mCherry/MYC) subgroup performed with the excel plugin MetaXL (V5.3). In both groups, no significant difference can be observed. Both display a high mean alpha/beta-ratio (11.01 +/- 5.78 and 13.56 +/- 3.34, respectively)

Interestingly, the TP53 genetic status had no influence on fractionation radiosensitivity (Table 1, ONS76 vs. all other cell lines). Although we analyzed a panel of distinct medulloblastoma cell lines, there was no relevant difference regarding low alpha/beta values detectable (*p* = 0.07) (Fig. 3). Furthermore, MYC expression also did not impact the fractionation sensitivity because neither in cells with aberrant baseline expression of MYC (Table 1, D283/D425 vs. all other cell lines) nor in cell lines genetically modified to overexpress MYC (Table 2, mCherry vs. MYC) and regardless of TP53 status (wild-type vs. mutated), a change from a high to a low alpha/beta value was observed (Fig. 3). This suggests that these genetic factors may not singularly govern the fractionation sensitivity profile of medulloblastoma.

**Fig. 4:**
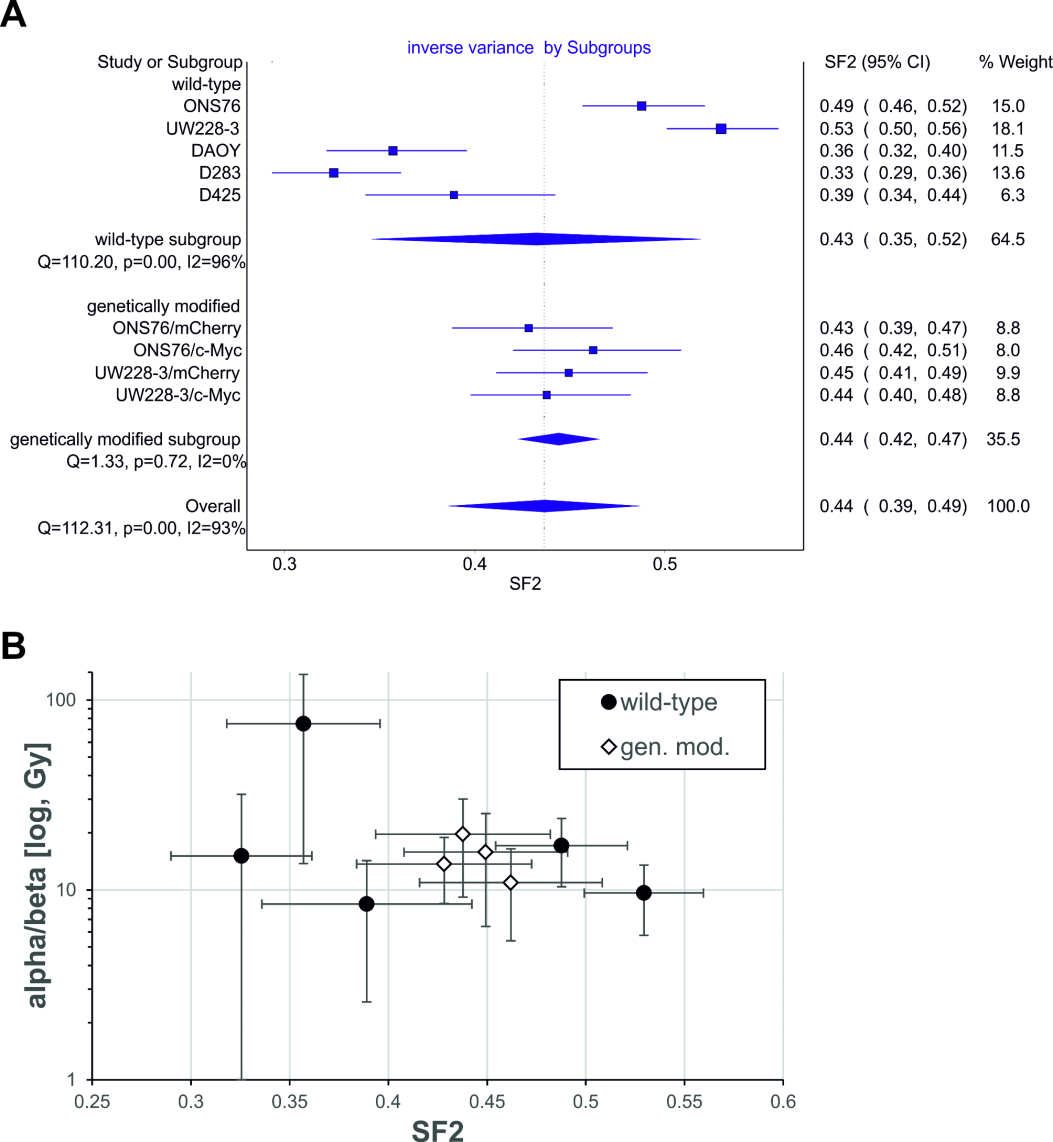
Graphical representation of the meta-analyses of the determined SF2-values in the tested medulloblastoma cell lines by MetaXL (**A**) Meta-analysis of the determined SF2-values in wild-type (ONS76, UW228-3, DAOY, D283, D425) and the genetically modified (ONS76/UW228-3 with mCherry/MYC) subgroup performed with the excel plugin MetaXL (V5.3). In the wild-type group, significant differences between the cell lines can be observed. (**B**) The calculated SF2 (x-axis) and alpha/beta values (y-axis) were plotted for every tested cell line of the wild-type (filled squares) and genetically-modified (open diamonds) subgroups. No correlation between these two parameters could be determined

The survival fraction of a single dose with 2 Gy (SF2) as an indicator of radiosensitivity was calculated using the LQ-model and the obtained values for the sensitivity parameters alpha and beta (Table 3). In the wild-type subgroup, significant differences between the medulloblastoma cell lines were observed, whereas the overexpression of c-Myc in the ONS76 or UW228-3 did not result in a change in radiosensitivity (Table 3; Fig. 4A). In this regard, the genetically modified cell lines displayed similar calculated survival rates after irradiation as their parental ONS76 and UW228-3 cells, respectively. In addition, radiosensitivity (SF2) did not correlate with fractionation sensitivity (alpha/beta) within all cell lines (Fig. 4B).

## Discussion

In this study, we determined alpha/beta values of medulloblastoma cell lines using formulas derived from the LQ-model of radiation survival with data obtained using the Well Control Dose Assay (WCD). The results indicate that all tested cell lines possess a high alpha/beta ratio, whereas mammary carcinoma MCF-7 cells display a low alpha/beta value. This finding demonstrates the ability of the WCD to distinguish between such cell lines, which suggests that medulloblastoma would be treated with hyperfractionation, whereas mammary carcinoma would be exposed to hypofractionated irradiation.

The classical approach to measure radiation survival is the colony forming assay (CFA). While many important discoveries were made using the CFA, its application for estimating alpha/beta values often results in very high confidence intervals with no real explanatory significance. In contrast, the WCD assay is a clonogenic survival assay very similar to the well established spheroid control assay that has been successfully used to estimate alpha/beta-values with narrow 95% confidence limits [17, 18]. Our approach with the WCD offers several experimental advantages over the CFA. First, we do not require single cells to successfully form a growing colony. Instead a steady population of cells with higher densities is seeded, allowing them to interact and therefore more reliably form a recurrence in a more physiological manner. In addition, the scoring of our data is binary: regrowth or no regrowth. Therefore it is much simpler and less subjective than counting up to hundreds of colony spots per single well when performing a CFA. In addition, these spots, when analyzed microscopically, often might not resemble a functioning cell clone (for an excellent protocol on how to perform the CFA and examples of the manifold pitfalls accompanied with it, see ref. [19]). Taken together, the WCD offers a more robust and reproducible experimental setup to estimate alpha/beta-values of cell lines with greater precision. However, it has its disadvantages. In contrast to the CFA, the WCD requires more preparatory work to establish correct cell numbers and radiation doses. In addition, its experimental setup is substantially more complex and involves a much longer readout time of up to 60 days. Because of this extended incubation times, cell lines that divide very slowly or grow semi-adherently can be very difficult to analyze accurately. However, cell lines with such characteristics would also pose a big challenge for the CFA.

To the best of our knowledge, this study reports the first in vitro data of alpha/beta values in cell lines derived from pediatric medulloblastoma patients. Normal and malignant tissues exhibit varying responses to fractionation, a phenomenon known as fractionation sensitivity. This sensitivity is typically described using the alpha/beta-ratio. Our preclinical data provide evidence that medulloblastomas in children exhibit high alpha/beta values, indicating reduced sensitivity to fraction size, independent of their overall sensitivity to radiation. This observation has been well-documented in adult head and neck malignancies, where administering radiotherapy in smaller fractions is known to better protect late-responding normal tissues in comparison to the tumor [20]. In the context of medulloblastoma, analogous clinical observations have substantiated our findings.

Hyperfractionated radiotherapy was implemented with the objective of maximizing the therapeutic dose to the tumor while simultaneously minimizing the risk for adverse effects on surrounding normal tissues. For low or standard-risk patients, where high cure rates are often achievable with current normofractionated concepts, the emphasis of hyperfractionated radiotherapy is on reducing long-term toxicities and thereby preserving neurological function and quality of life. In high risk patients, where the prognosis is poorer and the risk of disease recurrence is higher, the aim shifts towards enhancing the efficacy of radiotherapy without disproportionately increasing toxicity. However, the clinical experiences to date have not definitively demonstrated a significant advantage for hyperfractionated radiotherapy over conventional radiotherapy in terms of survival outcomes.

The HIT-SIOP PNET 4 trial (2001–2006) examined the role of hyperfractionation in comparison to conventional radiation therapy in the treatment of pediatric patients with medulloblastoma [21]. Despite the absence of a clear advantage in terms of 5-year event-free survival (78% for hyperfractionated vs. 77% for conventional) in the HIT-SIOP PNET 4 trial (2001–2006), hyperfractionated therapy did reveal a significant improvement in verbal intelligence quotient (VQ) among children under 8 years at diagnosis (mean intergroup difference 12.02, *P* = .02) and exhibited a noticeable trend toward enhanced processing speed (mean intergroup difference 10.90, *P* = .08) [22].

In the clinical application of hyperfractionated radiotherapy in young children, significant challenges exist, irrespective of radiobiological considerations [9]. For very young children, sedation for immobilization is necessary for treatment planning and delivery. The restrictions for both food and fluids before sedation pose substantial challenges for families. Although the feasibility of this approach has been demonstrated by the St. Jude Hospital group, the twice-daily sedation of young children remains a topic of controversy [23]. This approach requires substantial resources and its safety has not been conclusively established.

Controversial results have been reported for metastatic medulloblastoma, previously. While the Italian group demonstrated promising outcomes with Hyperfractionated Accelerated RT (HART) combined with pre-RT chemotherapy and maintenance chemotherapy for metastatic medulloblastoma, achieving a 5-year event-free survival (EFS) of 70%, attempts by the British to replicate these results using the same approach resulted in a lower 3-year EFS of 59% [10]. These disparities may be attributed to the heterogeneous radiobiological behavior of metastatic tumors [24].

Incorporating predictive molecular biomarkers for radiation sensitivity holds the potential to enhance the customization of radiotherapy to individualized conditions. Both the tumor suppressor TP53 as well as the proto-oncogene MYC have been described as important modulators of proliferation and cell survival after DNA damage [25–27]. However, in our hands both did not influence the SF2 in the two tested cell lines (ONS76 and UW228-3). In addition, irrespective on their proposed impact on radiosensitivity, our results reveal comparable outcomes regarding the fractionation sensitivity marker alpha/beta across different sub-classifications, surprisingly regardless of the presence or absence of MYC overexpression and/or TP53 alterations. Therefore, the consistency in radioresponse observed in our study suggests the potential feasibility of a standardized radiotherapeutic approach for medulloblastoma, irrespective of specific molecular profiles. While various cellular response mechanisms contributing to fractionation sensitivity have been identified in the past, the molecular biomarkers remain elusive.

Our investigations heavily rely on in vitro assessments conducted on tumor cell lines and it is important to recognize that these controlled laboratory conditions may not comprehensively replicate the intricate microenvironment encountered in clinical settings. The inherent limitations of this approach lie in its inability to encompass the full spectrum of variables that influence radiation sensitivity within the complex context of real-world patient scenarios. The Linear-Quadratic (LQ) model is widely used in radiotherapy to predict the biological response of tissues to radiation. However, it has several limitations:

The LQ model may not accurately predict cell survival at very low doses of radiation, where the linear component dominates. At high doses, the quadratic component can overestimate cell killing, making the model less reliable for high-dose treatments. The model assumes uniform radiosensitivity across different tissues and tumors, which may not reflect the true biological variability. It does not fully account for complex repair mechanisms and repopulation dynamics that occur in cells after radiation.

These limitations suggest that while the LQ model is useful, it should be applied with caution and supplemented with other models or clinical data when necessary.

However, our data can serve to support prior clinical observations and provide a foundation for establishing normo- to hyper-fractionated radiation protocols for young children with medulloblastoma, based on alpha/beta values. Nonetheless, we acknowledge that larger prospective randomized studies are highly desirable to further validate and refine these findings.

## Data Availability

The supporting data can be made available by the first and last authors.

## Abbreviations

α: Radiosensitivity parameter
β: Radiosensitivity parameter
CFA: Colony forming assay
CI: 95% confidence intervall
CSI: Craniospinal irradiation
d: Single dose
D: Total dose
DNA: Desoxyribonucleic acid
γ: Repopulation factor
Gy: Gray
EFT: Event free survival
k: Number of clonogenic cells
LQ: Linear quadric model
n: Number of fractions
RT: Radiotherapy
SF2: Surviving fraction after a single dose of 2 Gy
SHH: Sonic Hedgehog
t: Treatment time
WCD: Well control dose
WNT: Wingless-related integration site
WHO: World Health Organization
